# 2407. Multidisciplinary Endocarditis Team (MET): Establishing a Baseline of Infective Endocarditis in People Who Use Injection Drugs (PWID)

**DOI:** 10.1093/ofid/ofad500.2027

**Published:** 2023-11-27

**Authors:** Kaya Patel, Olivia Duffield, Stephanie Spivack, Sara K Schultz

**Affiliations:** Temple University Hospital, Philadelphia, Pennsylvania; Temple University School of Medicine, Philadelphia, Pennsylvania; Temple University Hospital, Philadelphia, Pennsylvania; Temple University Hospital, Philadelphia, Pennsylvania

## Abstract

**Background:**

Infective endocarditis (IE) is a serious infectious complication of intravenous drug use. Treatments include prolonged courses of IV antibiotics, surgical valve repair/ replacement, vacuum-assisted thrombectomy, and pulmonary autograft (Ross Procedure). We created a multidisciplinary team of treating physicians to review all cases of IE at our large academic medical center in Philadelphia starting in March 2023. To establish a baseline prior to the creation of MET, we describe the microbiological and clinical findings, treatment, and outcomes of a cohort of PWID found to have IE.

**Methods:**

We queried our EMR for admissions with patients that had documented opioid use disorder with injection behavior and positive blood cultures from September of 2021 to March of 2022. We conducted a retrospective chart review of these patients and collected data regarding their hospital course and treatment.

**Results:**

We identified 39 patients with definite IE (modified Duke’s criteria). The predominant organisms were MRSA (19, 43%), MSSA (14, 32%), and group A streptococcus (9, 20%). Thirty-six (92%) patients underwent TTE and 8 (20%) underwent TEE to evaluate for vegetations. On echocardiography, 23 (59%) vegetations were noted: 11 tricuspid valve, 3 mitral valve, 2 aortic valve, and 2 pulmonic valve. Five vegetations were found on prosthetic valves: 2 tricuspid valve, 2 pulmonic valve, and 1 aortic valve. Of the 23 patients with vegetations, 11 (48%) met ACC/AHA criteria for procedural intervention. Of these patients, 7 (63%) received intervention (1 valve repair, 2 valve replacements, 2 Ross Procedures, 2 vacuum-assisted thrombectomies). Intervention occurred on average of 7.8 days from diagnosis. Sixteen (41%) patients completed a full course of antibiotic therapy. Of the 23 (59%) patients that did not complete antibiotics, 5 were prescribed oral 2nd line therapies. Fifteen (38%) patients were readmitted within 90 days and 5 (13%) experienced mortality within 30 days. (Figure 1)

**Figure:**
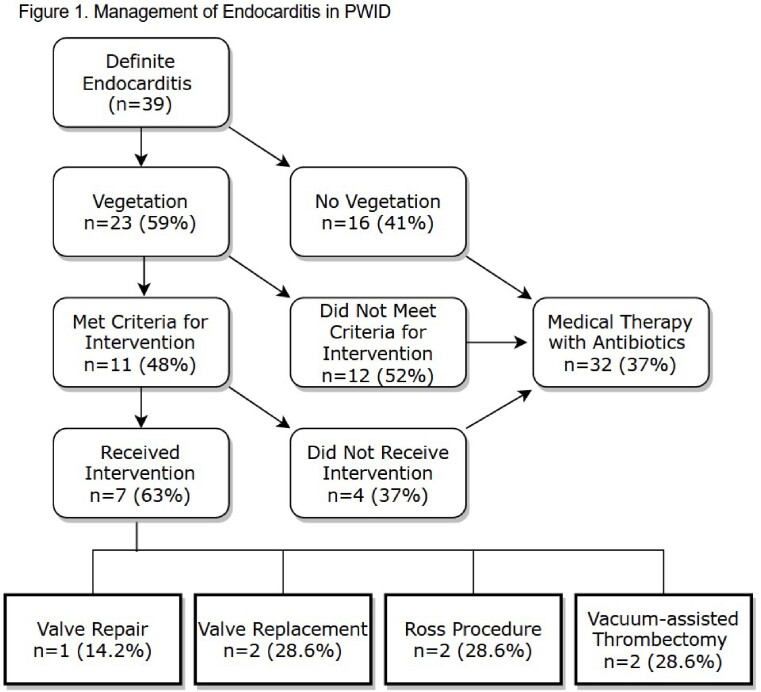

**Conclusion:**

Treating IE in PWID is challenging and often requires a multidisciplinary approach. We demonstrated high rates of right and left sided IE as well as high rates of surgical intervention pre-implementation of MET. We anticipate an improvement in clinical outcomes with the implementation of MET.

**Disclosures:**

**Sara K. Schultz, MD FACP FIDSA**, AbbVie: Advisor/Consultant

